# Health care transition for patients with vascular malformations: a French multicenter cross-sectional study

**DOI:** 10.1186/s13023-021-01970-7

**Published:** 2021-08-06

**Authors:** Camille Vermersch, Olivia Boccara, Christine Chiaverini, Juliette Mazereeuw-Hautier, Nina Sigg, Stéphanie Mallet, Pierre Vabres, Denis Herbreteau, Anne Le Touze, Annabel Maruani, Sophie Leducq, Research Group of the Société Française de Dermatologie Pédiatrique (French Society of Pediatric Dermatology)

**Affiliations:** 1 Department of Dermatology and Reference Center for Rare Diseases and Vascular Malformations (MAGEC), CHRU Tours, Avenue de La République, 37044 Tours Cedex 9, France; 2 Department of Dermatology and Reference Center for Genodermatoses and Rare Skin Diseases (MAGEC), Necker-Enfants Malades University Hospital, APHP5, Paris, France; 3 Imagine Institute, Paris University, Necker-Enfants Malades University Hospital, APHP5, Paris, France; 4 Department of Dermatology, University Hospital Center of Nice, Nice, France; 5 Department of Dermatology, University Hospital Center of Toulouse, Toulouse, France; 6 Department of Dermatology, University Hospital Center of Angers, Angers, France; 7 Department of Dermatology, University Hospital Center of Marseille, 13885 Marseille Cedex 5, France; 8 Department of Dermatology, University Hospital Center of Dijon, Dijon, France; 9 Department of Neuroradiology and Interventional Radiology, University Hospital Center of Tours, Tours, France; 10 Department of Pediatric Surgery, University Hospital Center of Tours, Tours, France; 11 Universities of Tours and Nantes, INSERM 1246-SPHERE, 37000 Tours, France

**Keywords:** Rare skin diseases, Health care transition, Patient transfer, Vascular malformations, Adult, Children, Survey, Patient satisfaction

## Abstract

**Background:**

Health care transition (i.e., transition from pediatric to adult care) is challenging in chronic conditions but has been poorly studied in rare chronic skin diseases. We investigated the proportion of lost to follow-up among patients with superficial vascular malformations after health care transition. We also collected patients’ opinions. This prospective, multicenter, cross-sectional study was performed at 7 French hospitals. We included patients aged 19–25 years, who were followed for a superficial vascular malformation before age 16, and who had completed the transition period in 2020. Data were collected from medical records and a questionnaire was sent to included patients asking about the health care transition.

**Results:**

Among the 90 patients included, 41 (46%) were lost to follow-up after health care transition period. The age at diagnosis was significantly higher for lost to follow-up than non- lost to follow-up patients. The lost to follow-up proportion was similar between patients who changed and did not change hospitals during the transition. Responses to the questionnaire were obtained for 47 of 90 patients (52.2% response rate); most were satisfied with their care (n = 31/36, 86.1%); however, a lack of psychological support was reported.

**Conclusions:**

Health care transition is associated to a high rate of lost to follow-up. Early management seems associated to less lost to follow-up. Further studies are needed to better understand risk factors for a failed health care transition and its consequences.

## Background

Superficial vascular malformations (SVMs) are congenital rare conditions of children and adults, with a prevalence estimated at 1.2% [1]. In numerous types of SVMs, post-zygotic mutations in genes involved in vasculogenesis have been identified; the consequences are inborn errors in vascular morphogenesis [2, 3]. SVMs are divided into four subtypes according to the vessels involved: capillary, lymphatic, venous and arteriovenous malformations, that can be isolated, combined, or associated with other anomalies [4]. Although SVMs are present at birth, they are not always apparent at this stage and might even manifest during adulthood [5]. The natural history of SVMs depends on the subtype and usually consists of progressive worsening during life which often leads to moderate to severe functional impairment and decreased quality of life [6–8]. Except for very small SVMs that have curative treatment, management of SVMs is a life-long process that requires long-term follow-up during childhood and adulthood.

Adolescence is a complex period for management because it involves multiple transformations. Hormonal changes may worsen SVMs [6]; also, the adolescents’ desire for independence might lead them to disrupt their treatment and follow-up, which also might induce disease worsening [9]. Finally, the health care systems, including caregivers, sometimes change during this transition period, depending on each country’s health care organization [10]. For example, in France, pediatric hospitals and health care units usually see children until they reach 15 years and 3 months, then adolescents are cared for in adult departments. This transition from pediatric to adult care is called “health care transition” (HCT). HCT can be defined as “the process of moving from a pediatric to an adult model of health care with or without a transfer to a new health care provider” [11]. HCT for patients with a chronic disease requires providing “health care that is uninterrupted, coordinated, developmentally appropriate, psychosocially sound, and comprehensive” [12]. It has been described and analyzed in chronic pathologies such as HIV or diabetes [13, 14]. However, in rare diseases, HCT is all the more challenging as there is a strong relationship between the patient and the physician due to long-term follow-up, and also it may be difficult to find physicians with a good knowledge of the disease in adult departments. A recent literature review of HCT in 2020 highlighted practical considerations to facilitate the development of programs for epidermolysis bullosa and few other dermatologic conditions [15], still there are no published data on HCT in rare chronic conditions such as SVMs. Although they do not threaten life as for HIV or diabetes, a successful transition care is necessary to ensure regular appointments during adulthood for optimal management.

The objectives of this study were to (1) assess the prevalence of lost to follow-up (LFU) in patients with SVMs during HCT; (2) evaluate the impact of HCT on care consumption; (3) describe modalities of HCT in French centers and (4) describe patients’ perception on HCT.

## Results

### *Characteristics of study participants* (Table 1)

**Table 1:** Characteristics of patients included

	Missing data	N = 90
Demographic characteristics		
Age, years	2	21.0 [20.0–22.25]
Female sex	0	51 (56.7)
Clinical features of vascular malformation		
Vascular malformation type	0	
Simple venous malformation		38 (42.2)
Simple lymphatic malformation		12 (13.4)
Simple arteriovenous malformation		9 (10.0)
Combined malformation (capillary/venous/lymphatic)		20 (22.2)
Vascular malformation associated with other anomalies		11 (12.2)
Location*	1	
Head and neck/tongue		30 (33.3)
Trunk/gluteal area		22 (24.4)
Upper limb		16 (17.8)
Lower limb		36 (40.0)
Age at diagnosis	10	3.0 [0.0–11.0]
Treatment of malformation*	0	
Therapeutic abstention		5 (5.6)
Sclerotherapy/embolization		42 (46.7)
Drugs		34 (37.8)
Physical treatment (bandages, drainage, laser)		31 (34.4)
Surgery		44 (48.9)
Medical data puberty		
Age at first period (for women only)	7	13.0 [12.0–14.0]
Age at growth spurt	65	13.0 [12.0–14.0]
Aggravation of the malformation in puberty (reported in the medical record)	7	35 (38.9)
Aggravation of the malformation in puberty (reported by the patient)	43	35 (74.5)

Results are n (%) for categorical variables and median [interquartile range] for quantitative variables

*A patient may have several characteristics

**Fig. 1:**
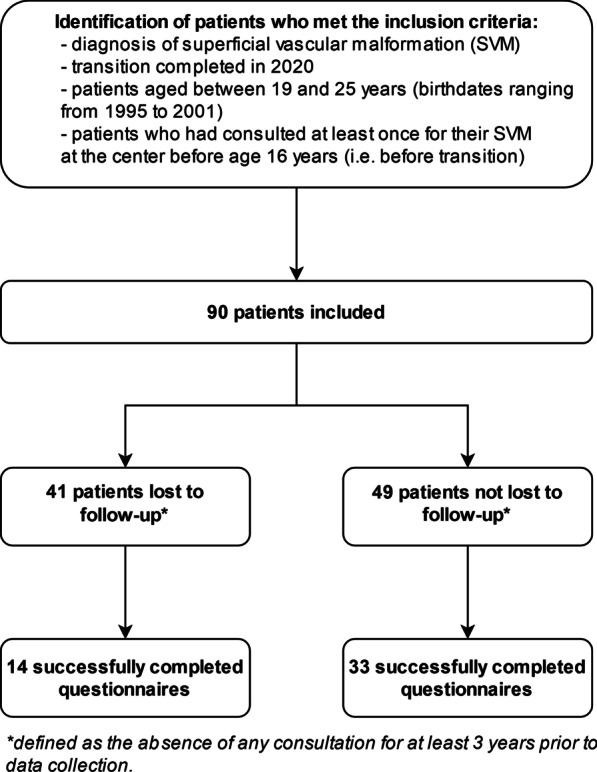
Flow of participants in the study

A total of 90 patients were included (Fig. 1): 51 (56.7%) women and 39 men (43.3%). The median age was 21.0 years (interquartile range 20–22.25 [range 19–25 years]). All types of SVMs were represented, most being venous malformations (38 patients, 42.0%). The most frequent location was the lower limb (n = 36, 40.0%). The median age at diagnosis was 3.0 years (interquartile range 0.0–11.0). In total, 85 patients had previous treatment. For 35 (38.9%), disease worsening during puberty was reported in medical records.

### Primary outcome

Altogether, 41 (45.6%) patients were LFU during HCT. Table 2 shows the characteristics of patients by LFU status. Age at diagnosis was significantly higher in patients LFU than not LFU (9.0 vs 2.0 years, *p* = 0.038). The groups did not differ in age, sex, age of onset of follow-up in the hospital site, vascular malformation type, previous treatment of the malformation, frequency of physician’s visits and need to change hospital site (from pediatric department to adult department).

**Table 2:** Patient characteristics by their loss to follow-up status

	Missing data	Lost to follow-up (N = 41)	Not lost to follow-up (N = 49)	*p* value
**Demographic characteristics**				
Age, years	2	21.0 [20.0–22.25]	21.0 [20.0–22.25]	0.42
Female sex	0	25 (61.0)	26 (53.1)	0.28
**Clinical features of vascular malformation**				
Vascular malformation type	1			0.059
Simple venous malformation		23 (56.1)	15 (30.6)	
Simple lymphatic malformation		7 (17.1)	6 (10.2)	
Simple arteriovenous malformation		2 (4.9)	7 (14.3)	
Combined malformation (capillary/venous/lymphatic)		6 (14.6)	14 (28.6)	
Syndromic malformation		3 (7.3)	8 (16.3)	
Location*	1			0.53
Head and neck/tongue		10 (24.4)	20 (40.8)	
Trunk/gluteal area		10 (24.4)	12 (24.5)	
Upper limb		8 (19.5)	8 (16.3)	
Lower limb		18 (43.9)	18 (36.7)	
Age at diagnosis	10	9.0 [0.0–13.0]	2.0 [0.0–6.5]	**0.038**
Previous treatment of malformation*	0			0.055
Therapeutic abstention		4 (9.8)	1 (2.0)	
Sclerotherapy/embolization		18 (43.9)	24 (49.0)	
General drug treatment		7 (17.1)	27 (55.0)	
Physical treatment (bandages, drainage, laser)		13 (31.7)	18 (36.7)	
Surgery		14 (34.2)	30 (61.2)	
**Follow-up procedures in the center**				
Age at onset of follow-up in the site	2	13.0 [9.0–14.0]	9.0 [3.5–13.0]	0.07
Type of site	0			0.71
Pediatric site		30 (73.2)	38 (77.5)	
Adult site		1 (2.4)	0 (0.0)	
Mixed site		10 (24.4)	11 (22.5)	
Transition/change of site during follow-up (from pediatric to adult department)	1	9 (22.5)	14 (28.6)	0.23
Age at change of site	2	17.0 [16.0–16.5]	16.0 [14.5–16.5]	0.09
Average number of consultations^†^	7			0.08
≤ 1 time per year		11 (26.8)	25 (51.0)	
> 1 time per year		30 (73.2)	24 (49.0)	

Significance of bold:* p* < 0.05 was considered statistically significant

Results are n (%) for categorical variables and median [interquartile range] for quantitative variables

*A patient may have several characteristics

^†^Mean number of consultations per year corresponding to the total number of consultations/number of years of follow-up

### Health care consumption

Table 3 displays the health care consumption by age during HCT for patients during follow-up. Scheduled consultations were from 0 to 10 per year for each age from pre-transition (age 13 years) to 19 years, with no increase in frequency during follow-up. Unscheduled hospitalization and admission to the emergency department ranged from 0 to 2 and 0 to 3 per year, respectively, for each age and were similar between age ranges.

**Table 3:** Health care consumption during follow-up and health care transition by age range (from pre-transition [age 13 years] to 19 years)

	Missing data	13–14 years	14–15 years	15–16 years	16–17 years	17–18 years	18–19 years
Patients with > 1 scheduled consultations	5	13/70 (18.6%)	27/76 (35.5%)	16/70 (22.9%)	19/66 (22.8%)	24/62 (38.7%)	23/56 (41.1%)
Number of scheduled consultations, median [range]	5	0 [0–6]	0 [0–5]	1 [0–6]	1 [0–5]	1 [0–10]	1 [0–10]
Patients with ≥ 1 emergency department visit(s)	5	0/70 (0.0%)	4/76 (5.3%)	1/71 (1.4%)	1/67 (1.5%)	3/63 (4.8%)	2/57 (3.5%)
Number of emergency department visits, median [range]	5	0 [0–0]	0 [0–2]	0 [0–3]	0 [0–1]	0 [0–1]	0 [0–2]
Patients with ≥ 1 unscheduled hospitalization(s)	5	0/72 (0.0%)	2/78 (2.6%)	1/73 (1.4%)	0/69 (0.0%)	2/65 (3.1%)	1/59 (1.7%)
Number of unscheduled hospitalizations, median [range]	5	0 [0–0]	0 [0–2]	0 [0–2]	0 [0–1]	0 [0–1]	0 [0–2]

Results are number/number (%) of patients at risk for the period and median [range]

We defined health care transition-age as ranging from 13 to 19 years old, because at 13 years old, the transition to adult care has not yet begun and at 19 years, this process is considered completed

### Modalities of HCT in participating French departments

Among the 7 French tertiary centers that participated to the study, health providers were the same for children and adults followed up for SVMs in 6 centers and different in one. In 5 centers, adult and pediatric consultations took place at the same hospital site, and for 2 centers, patients had to change hospitals (from a specific pediatric one to an adult one) during the HCT. One center had a professional HCT coordinator and one center offered support groups or therapeutic workshops. The center with the HCT coordinator had similar results than the centers without HCT regarding number of loss to follow up (n = 9/14 patients LFU vs n = 32/76 patients LFU).

### Patients’ perceptions of the transition process

Among all 90 patients, 47 completed the questionnaire (52.2% response rate): 14 were LFU from the hospital center (29.8%), and 33 were still followed up (70.2%) (Fig. 1). The characteristics of responding patients are in Table 4. The median age of the responders was 21.0 years (interquartile range 20–22.0), 29 (61.7%) were females (i.e., male/female ratio 1:1.6); 36 (76.6%) were in college and 6 (12.8%) had a professional activity. LFU and non-LFU patients did not differ in change of city for studies, discomfort linked to the malformation during adolescence and rate of patients who wanted to interrupt their follow-up during adolescence. Most patients were satisfied with their care (n = 31, 86.1%; missing data for 11 patients), with no difference between LFU and non-LFU groups. However, a lack of psychological support was reported (only 5 patients who responded to the questionnaire had been followed by a psychologist).

**Table 4:** Characteristics of responders to the survey regarding their loss to follow-up status

	Missing data	Total (N = 47)	Lost to follow-up (N = 14)	Not lost to follow-up (N = 33)	*p* value
**Demographic characteristics**					
Age, years	0	21.0 [20.0–22.0]	20.5 [20.0–22.0]	21.0 [20.0–22.0]	0.58
Female sex	0	29 (61.7)	13 (92.9)	16 (48.5)	**0.01**
Professional status					0.19
High school		1 (2.1)	0 (0.0)	1 (3.0)	
College		36 (76.6)	10 (71.4)	26 (78.8)	
Professional activity		6 (12.8)	1 (7.1)	5 (15.2)	
Not working because of disability		2 (4.3)	2 (14.3)	0 (0.0)	
Not working		2 (4.3)	1 (7.1)	1 (3.0)	
Education level	1				0.97
Primary/high school education		25 (54.3)	7 (53.9)	18 (54.5)	
College/university education		21 (45.7)	6 (46.1)	15 (45.5)	
Marital status	0				> 0.99
Single		42 (89.4)	13 (92.9)	29 (87.9)	
Married/common law		5 (10.6)	1 (7.1)	4 (12.1)	
Change of city for studies	0	25 (53.2)	7 (50.0)	18 (54.5)	> 0.99
**Data on the period of adolescence/transition**					
Embarrassment or complex due to malformation	1				> 0.99
Yes, absolutely		20 (42.5)	6 (46.2)	14 (42.4)	
Yes, rather		10 (21.7)	3 (23.0)	7 (21.2)	
No, not really		8 (17.4)	2 (15.4)	5 (15.2)	
Not at all		8 (17.4)	2 (15.4)	6 (18.2)	
Want to stop follow-up	1				0.72
Yes, absolutely		7 (15.2)	3 (23.1)	4 (12.1)	
Yes, rather		7 (15.2)	2 (15.4)	5 (15.2)	
No, not really		9 (19.6)	3 (23.1)	6 (18.2)	
Not at all		23 (50.0)	5 (38.4)	18 (54.5)	
Patients satisfied with follow-up	11	31 (86.1)	8 (88.9)	23 (85.2)	> 0.99
Regularity of appointments	13				> 0.99
Adapted		25 (75.7)	6 (75.0)	19 (79.1)	
Not enough		6 (15.2)	1 (12.5)	5 (20.8)	
Too frequent		3 (9.1)	1 (12.5)	2 (8.3)	
Psychological follow-up	1	5 (10.6)	1 (7.1)	4 (12.5)	> 0.99
Change of site during monitoring	1	22 (47.8)	9 (69.3)	13 (40.6)	0.20
Age at change of site	2	16.0 [16.0–17.5]	16.0 [15.5–17.5]	16.0 [16.0–17.8]	
Decision to change site	3				> 0.99
Good time		16 (84.2)	7 (87.5)	9 (81.8)	
Too soon		3 (15.8)	1 (12.5)	2 (18.2)	
Too late		0 (0.0)	0 (0.0)	0 (0.0)	
Change in support	0				0.29
Yes, absolutely		3 (13.6)	1 (11.1)	2 (15.4)	
Yes, rather		7 (31.8)	5 (55.6)	2 (15.4)	
No, not really		7 (31.8)	2 (22.2)	5 (38.4)	
Not at all		5 (22.7)	1 (11.1)	4 (30.8)	
**Data for the current (post-transition) period**					0.66
Embarrassed or annoyed by the malformation	0				
Yes, absolutely		12 (25.5)	3 (21.4)	9 (27.3)	
Yes, rather		9 (19.1)	2 (14.3)	7 (21.2)	
No, not really		8 (17.0)	4 (28.6)	4 (12.1)	
Not at all		18 (38.3)	5 (35.7)	13 (39.4)	

Significance of bold:* p* < 0.05 was considered statistically significant

Results are n (%) for categorical variables and mean ± SD or median [interquartile range] for quantitative variables

## Discussion

To our knowledge, this is the first study to describe the HCT process in patients with SVMs. Among 90 patients included, 41 (45.6%) were LFU during transition period. The change in hospital site (from pediatric to adult department) during the transition was not associated with increased frequency of LFU. Age of diagnosis of SVMs was significantly higher for patients LFU than non-LFU (*p* = 0.038).

There are many challenges inherent in the transition process from pediatric to adult care. This critical period takes place during adolescence, which is associated with physical and psychosocial changes, with a risk of SVM worsening, as reported for 39% of our patients. As a consequence, we could have expected an increased number of consultations and a low rate of LFU, but our study highlights a high rate of LFU (45.6%). This could be explained by several factors: lack of curative treatments [16–18], prevalence of the disease, overloaded school schedules, follow-up fatigue in adolescents, or opposition to parents, for example. Indeed, in more common conditions (HIV and type 1 diabetes), the LFU rate is lower (19.8% and 8.6%, respectively) and could be explained by the long-term treatment [19, 20] and because these are life-threatening diseases. Recently, sirolimus, a mammalian target of rapamycin inhibitor, has been increasingly used for complicated vascular malformations [21], and the targeted PIK3CA inhibitor alpelisib (BYL719) was recently administered with significant clinical benefit in SVMs in the PIK3CA-related overgrowth spectrum [22]. The prospect of new effective drugs for VMs could help reduce the number of LFU.

In our study, early diagnosis was associated with decreased risk of LFU. We can assume that when the diagnosis is early, the child's and parents' confidence in the medical team and therefore adherence to follow-up is better. Also, more extensive and therefore more troublesome SVMs may lead parents to consult earlier. This finding might be linked to the following: (1) patients with regular appointments during childhood might continue these into adulthood and (2) patients with a late first appointment probably have a less serious form that does not require treatment and for which regular follow-up is not required. Furthermore, we highlight that a transition coordinator was present in only one of the 7 centers. For other chronic conditions, the presence of a transition coordinator, who helps coordinate patient intake and follow-up as well as answers questions/concerns that the patient or family may have, seems an important element for the success of the process [23–25]. Contrary to diabetes, for example, SVMs are rare chronic conditions, and therefore a small number of patients receive treatment in each center. The implementation of transition protocols for small numbers of patients can be more difficult.

In our study, consumption of scheduled care from pre-transition (age 13 years) to 19 years was higher than unscheduled care. Rates of scheduled consultations seem similar to data from a Canadian study finding 44.3% of patients with non-complex chronic conditions consulting a specialist in the 2 years after HCT [26]. Rates of hospitalizations were higher than in this study, which found a rate of 6.3% of hospitalizations in the 2 years after HCT. In our study, consumption of unscheduled care was very low, with very few admissions to the emergency department or unplanned hospitalizations. In the previously mentioned study [26], rates of admissions to the emergency department were much higher: 45.9% of patients admitted in the 2 years after HCT.

The survey part of our study highlighted an important psychological impact of the SVM as reported by the patient (42.5% of respondents were very embarrassed or annoyed by the malformation). The satisfaction with psychological care by their doctor was low. Very few patients had a follow-up by a psychologist (10.6%). Rates of psychological follow-up during this period were higher with other pathologies such as tuberous sclerosis complex (35.0%) [27].

We did not evaluate adolescent medicine, including adolescent sexual and reproductive health, drug and alcohol use and abuse, smoking etc. in our study. Physicians should provide, during and after HCT, teaching and counseling in these areas, especially for patients with a chronic condition with potential psychological impact [28].

### Limitations

Our study may be underpowered because of the small sample size (N = 90), which is explained by the rarity of SVMs and the small number of centers included (n = 7). Second, the number of patients LFU might be overestimated, if the patient moved and was followed up in a hospital that did not participate in the study. Moreover, our data collection may have been incomplete because we did not have access to the care consumption of patients outside the center (attending physician, emergency room or dermatology department in another hospital). Last, the age of HCT in France is 15 years and 3 months, younger than in the US at 18 years of age. Results may vary for different ages of transition.

## Conclusions

For young people with SVMs, HCT is a crucial period with the hormonal and psychological changes of adolescence and the potential change of hospital. Early diagnosis and follow-up, associated with psychological care and periodic physician visits, was associated with less LFU during and after the HCT. Evaluating HCT with measurable factors to be able to improve practice is an ambitious but necessary goal in rare chronic diseases.

## Methods

### Study design and setting

We conducted a cross-sectional multicenter study in patients with SVMs. Seven French centers involved in the network of the reference centers for vascular anomalies participated: departments of dermatology/pediatric dermatology of Angers, Dijon, Marseille, Nice, Paris-Necker, Toulouse and Tours.

### Participants

We included all patients followed up for an SVM who had completed the transition period in 2020 (i.e., age 19–25 years old [birthdates from 1995 and 2001]). Criteria for eligibility included patients consulting at least once for their SVM at the center before age 16 years (i.e., patients who had not completed transition process) so we could investigate the transition period.

Patients all had an SVM (lymphatic, venous or arteriovenous malformations) confirmed by imaging (ultrasonography, CT-scan, or MRI) that did not have curative treatment. The SVM was simple, combined or associated with other anomalies, whatever the topography. We excluded patients with simple capillary malformations, because this condition might not always require systematic follow-up, and those with incomplete medical records.

### Definition of HCT age

There is no precise definition of HCT age, which varies in the literature [10] and depends on several factors, mostly the country hospital structure policies. In France, the age of transfer (physical change of hospital site from pediatric to adult department) is 15 years and 3 months. We assume that the adolescence period includes a “pre-transition” process, ranging from approximately age 13–16 years, then a transition process (including transfer), which starts from age 16 years and is usually completed at age 19 years and is followed by a post-transition period. Therefore, we decided to include patients who consulted at least once for their superficial vascular malformation (SVM) at the hospital center before age 16 years in order to include patients who had not completed their transition.

### Data collected

We collected the following data from clinical records: demographic characteristics (age, sex), characteristics of the SVM (type, localization, simple or combined), history (age of diagnosis, previous treatment, effect of puberty on the SVM), current follow-up and change of hospital site during follow-up, and care consumption (last pediatric/adult care; number of physician visits, hospitalizations and emergency department admissions from pre-transition (age 13 years) to 19 years.

Included patients were asked to complete a standardized questionnaire. To optimize the response rate, patients who did not complete the questionnaire were contacted by telephone**.** The questionnaire included 32 items respecting anonymity: (1) socio-professional data (age, sex, marital status, professional status, change of city for studies and education level); (2) data on the period of adolescence (first menstrual period, age of growth spurt, embarrassment or complex due to malformation, aggravation of the malformation during puberty); (3) data on medical care during the transition from pediatric to adult health care (change of hospital site during transition, desire to stop the follow-up, psychological follow-up, hospital follow-up); (4) data on satisfaction of the patient with the follow-up during adolescence/transition (satisfaction with follow-up and regularity of appointments); and (5) data for the current follow-up (embarrassment or complex due to malformation). No data on LFU status were collected from the questionnaires. Finally, a form was completed by the investigator of each center to collect data on the center's practices regarding modalities of HCT.

### Endpoints

The primary outcome was the proportion of patients who were LFU, defined as lack of any consultation for at least 3 years in the hospital site participating in the study before data collection. These data were collected from clinical records. Secondary outcomes were (1) the health care consumption, assessed by the number of hospitalizations, admissions to the emergency department and physician’s visit from pre-transition (age 13 years) to 19 years; (2) patients’ perception of HCT (by the questionnaire); and (3) modalities of HCT in French tertiary centers.

### Statistical analysis

Descriptive data are presented as median (interquartile range) or range for quantitative data and frequency (percentage) for categorical data. We used no methods to handle missing data. Means were compared by Student *t* test and Wilcoxon sum rank test when necessary. Frequencies were compared by chi-square test and Fisher’s exact test as appropriate. No sensitivity analysis was performed. Statistical analyses involved using R v4.0.2. Two-sided *p* < 0.05 was considered statistically significant.

## Data Availability

The datasets used and/or analysed during the current study are available from the corresponding author on reasonable request.

## Abbreviations

HCT: Health care transition
LFU: Lost to follow-up
SVM: Superficial vascular malformation
